# Plasticized PVC Membrane Modified Electrodes: Voltammetry of Highly Hydrophobic Compounds

**DOI:** 10.3390/membranes10090202

**Published:** 2020-08-27

**Authors:** Ernő Lindner, Marcin Guzinski, Bradford Pendley, Edward Chaum

**Affiliations:** 1 Department of Biomedical Engineering, The University of Memphis, Memphis, TN 38152, USA; bpendley@memphis.edu; 2 Vanderbilt Eye Institute, Vanderbilt University Medical Center, Nashville, TN 37232, USA; marcin.guzinski@vumc.org (M.G.); edward.chaum@vumc.org (E.C.)

**Keywords:** chemically modified electrodes, membrane-coated voltammetric sensors, antidepressant and immunosuppressant drugs, detection limit, resolution

## Abstract

In the last 50 years, plasticized polyvinyl chloride (PVC) membranes have gained unique importance in chemical sensor development. Originally, these membranes separated two solutions in conventional ion-selective electrodes. Later, the same membranes were applied over a variety of supporting electrodes and used in both potentiometric and voltammetric measurements of ions and electrically charged molecules. The focus of this paper is to demonstrate the utility of the plasticized PVC membrane modified working electrode for the voltammetric measurement of highly lipophilic molecules. The plasticized PVC membrane prevents electrode fouling, extends the detection limit of the voltammetric methods to sub-micromolar concentrations, and minimizes interference by electrochemically active hydrophilic analytes.

## 1. Introduction

**Scheme 1:**
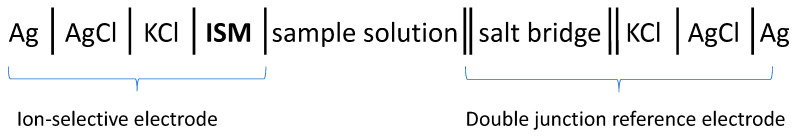
Electrochemical notation of a potentiometric cell with a conventional, liquid inner contact ion-selective membrane electrode as indicator electrode and a double junction reference electrode.

**Scheme 2:**
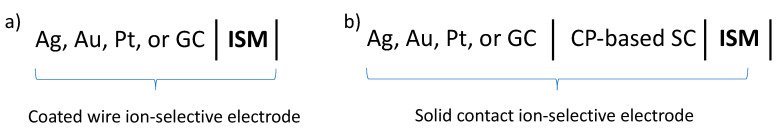
Electrochemical notation of solid contact ion-selective electrodes. (**a**) Coated wire electrode; (**b**) Conductive polymer (CP)-based solid contact electrode.

**Scheme 3:**
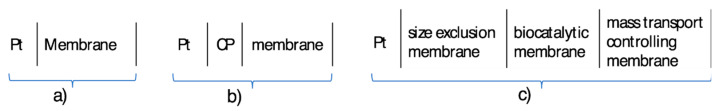
Electrochemical notation of membrane modified voltammetric working electrodes. (**a**) Membrane directly deposited on the substrate electrode surface; (**b**) A conductive polymer (CP) layer is sandwiched between the substrate electrode and the membrane; (**c**) Multiple membrane layer modified electrode.

There are a few areas of analytical chemistry where conventional methods have been almost completely displaced by electrochemical sensor-based measurements [1,2]. In these areas, the electrochemical sensors were integrated into fully automated clinical laboratory analyzers, e.g., ion-selective electrodes (ISEs), and/or incorporated into hand-held instruments for short turnaround time (STAT) point of care testing (POCT) devices, e.g., sensors for measuring blood glucose concentrations [3]. The most essential parts of the sensors used in these applications are unique membranes that, beyond individual measurements in small sample volumes, also permit interference-free continuous monitoring of essential analytes in complex biological matrices like sweat, urine and whole blood [4,5]. However, the role and function of the membranes utilized in the various sensors are often fundamentally different. For example, in potentiometric ISEs, the membrane is the sensing element of the sensor that provides the analytically relevant, concentration-dependent signal [6]. In contrast, in voltammetric sensors, various membranes and coatings are used to enable or improve the analytical signal of a Pt, Au or glassy carbon (GC) working electrode. The latter is the concept of chemically modified electrodes (CMEs) [7,8]. By selecting the proper modifications, desirable properties (e.g., selectivity or detection limit) may be achieved, and electrode fouling can be prevented. The goal of this mini-review is to show the evolution of a new class of voltammetric sensors with unique selectivities and exceptional detection limits that are based upon sensor structures and membranes, which are most commonly utilized in solid contact ISEs. In line with this goal, we first describe the structure of membrane separated, conventional electrochemical cells with symmetric and asymmetric contacts that are used for potentiometric measurements of anions and cations (Scheme 1 and Scheme 2). Next, we show the similarities in the layer structure of these electrochemical cells and cells utilizing membrane-coated working electrodes for the measurement of redox-inactive cations and anions by ion transfer voltammetry (Scheme 3b). Finally, through selected examples, we demonstrate that the membrane coating on the working electrode surface can be utilized for the selective extraction and simultaneous measurement (oxidation or reduction) of highly hydrophobic, electrochemically active compounds (Scheme 3a).

In conventional potentiometric sensors, the sensing membrane separates two solutions, the sample and the filling solution of the sensor, and the membrane potential is measured at zero current between two reference electrodes on the opposite sides of the membrane (Scheme 1). In 1971, Cattrall and Freiser [9] designed a simple compact electrode by dip-coating a Pt wire with ion-selective membrane (ISM) (Scheme 2a, coated wire electrode). Since the interface between the electron-conducting metal and the ion-conducting membrane in coated wire-type electrodes is not thermodynamically well defined (blocked interface) [10], these electrodes often have significant drift. The drift may also be related to the detachment of the membrane from the metal electrode surface and the formation of a thin water layer between the substrate electrode and the membrane [11,12]. To overcome some of the deficiencies of the coated wire electrode, an ion-to-electron transducer layer, generally a conductive polymer (CP) layer, has been sandwiched between the electron-conducting substrate and the ISM (Scheme 2b, solid contact ion-selective electrodes). In solid contact ISEs, the ISM is deposited directly over the conductive polymer by drop-casting or spin-coating (double polymer membrane modified electrodes). In electrochemical cells utilizing solid contact ISEs, the membrane potential is measured between the solid contact and the external reference electrode placed in the sample solution. The electrochemical notation of solid contact ISEs is shown in Scheme 2. In Scheme 1 and Scheme 2, ISM represents the ion-selective membrane, CP stands for conductive polymer, vertical lines represent phase boundaries, and two vertical lines represent a liquid/liquid junction. Under ideal conditions, each phase boundary is in thermodynamic equilibrium and the composition of the contacting phases, including the ion-selective membrane (ISM), is constant [6].

The layer structure of membrane-modified voltammetric sensors can be the same as that of solid contact ion-selective electrodes (Scheme 3a,b) or significantly more complex (Scheme 3c). The more complex layer structure is needed to enable the voltammetric sensors to be used for quantitative measurements in complex matrices. Although the oxidation and reduction of organic molecules on electrode surfaces offer almost limitless possibilities for both batch [13] and flow-analytical [14,15] measurements for a variety of analytes, beyond some exceptions, voltammetric sensors are mainly used in research laboratories [16]. The limited spread of practical analytical methods based on voltammetric sensors can be traced back to three main causes: (i) limited selectivity of voltammetric measurements and the difficulty of eliminating the influence of interfering compounds on the voltammetric signal; (ii) inadequate detection limit of the voltammetric methods and the challenges of quantification of analytes at sub-micromolar concentration; (iii) inconsistency of the voltammetric signals due to electrode fouling. Since the voltammetric signal (the measured current) is a function of the working electrode surface area, contaminations of the electrode surface from the sample or as a consequence of the electrochemical reactions may contribute to a negative or positive bias in the analytical signal. When resistive layers build up on or around the sensing surface, the sensor may lose its utility because its signal deteriorates and/or its response slows down. The buildup of resistive layers and encapsulation of sensors is most relevant during in-vivo applications when the sensors are implanted into tissues for monitoring. The issue is related to the biocompatibility of the sensors and the “sensor compatibility” of the environment where it is working [16,17,18]. While unwanted precipitation or adsorption processes may reduce the response of a voltammetric working electrode, targeted manipulation of the surface chemistries can enhance its electrochemical response, e.g., improve the selectivity or detection limit, prevent electrode fouling, etc. However, the “proper modifications” may require multi-layer constructions, as shown in Scheme 3b,c. For example, in ion-transfer voltammetry, double polymer membrane modified electrodes are used, [19,20,21] and the Pt working electrode surface of the voltammetric enzyme sensor for the measurement of glucose in whole blood is generally coated by a selectivity enhancing layer, a biocatalytic layer and a mass transport controlling layer. Each of these layers have specific roles for adequate voltammetric response.

**Figure 1:**
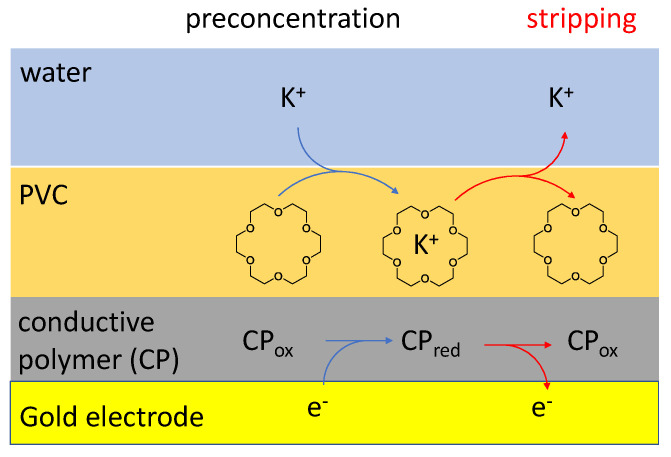
Schematic representation of the processes during the concentration (black arrows) and stripping (red arrows) of cations using a conductive polymer/plasticized PVC membrane modified electrode. The 18-crown-6 molecule in the PVC membrane represents any selective ionophore which may be used in this arrangement.

Within the large family of CMEs, voltammetric working electrodes with an organic film coating on their surfaces represent a special group. When the aqueous phase (sample) contains hydrophilic electrolyte and the organic film is loaded with a hydrophobic electrolyte, then the organic film/sample solution interface behaves analogously to polarizable working electrodes which can be used to drive ions across the aqueous/organic interface. The analytical signal is the current related to the ion-transfer of the measured ions across the aqueous/organic interface. The analytical methods utilized in combination with double membrane-coated working electrodes (Scheme 3b) evolved from ion-transfer voltammetry at the interface between two immiscible electrolyte solutions (ITIES) [20]. ITIES was developed for the measurement of redox-inactive, generally hydrophilic anions and cations [22,23,24], but has also been utilized for the voltammetric ion transfer of biologically relevant polyions such as heparin [25,26] and protamine [27]. ITIES is attractive because it enables the measurements of ions without their electrolysis [20,28]. With the double membrane arrangement, i.e., a conductive polymer-supported thin plasticized PVC membrane, as shown in Scheme 2b, the oxidation or reduction of the conductive polymer drives the transfer of anions or cations into the PVC membrane, respectively (Figure 1). The selectivity of the method is related to differences in the solvation energies of the different ions which can be facilitated by incorporating selective ionophores and or ion-exchange sites in the polymeric membranes [22,23,24] and enhanced by kinetic effects of ion hydrophobicity [29]. Using an adequate applied potential, the analyte ions can be concentrated in the organic phase. Following concentration, the ions can be stripped from the organic phase into the aqueous phase using a voltammetric technique, e.g., linear sweep voltammetry. By using a highly plasticized, ionophore loaded ion-selective membrane as the organic phase over a conductive polymer layer for ITIES, as in Scheme 2 and Figure 1, in combination with stripping voltammetry, subnanomolar concentrations can be measured for both cationic [30] and anionic [21,23] analytes.

In contrast to the ion-transfer voltammetric methods, the single membrane-coated working electrode (Scheme 3a) is used for the measurement of electrochemically active highly lipophilic analytes [31,32]. The highly lipophilic analytes spontaneously partition into the membrane where their oxidation/reduction provides the analytical signal. Consequently, these measurements can be considered as voltammetric determinations in a non-aqueous phase, i.e., within the membrane coating. Accordingly, potential sweep and potential step methods, with or without the integration of the recorded current, can all be used in combination with this electrode. The selectivity and detection limit of this working electrode is related to the membrane/solution partition coefficient of the analyte and interfering compounds. 

## 2. Materials and Methods

### 2.1. Membranes and Membrane Deposition

For the preparation of membranes poly(vinyl chloride) (PVC, high molecular weight), and bis(2-ethylhexyl)sebacate (DOS) were purchased from Sigma-Aldrich, St. Louis, MO, USA. Tetradodecylammonium tetrakis(pentafuorophenyl)borate (TDDA-TPFPhB) was prepared by metathesis reaction between tetradodecylammonium chloride (TDDACl) (Sigma-Aldrich, St. Louis, MO, USA) and high purity sodium tetrakis-(pentafluorophenyl)borate (KTPFPhB) (Boulder Scientific Company, Mead, CO, USA) in dichloromethane. PVC membrane solutions were prepared by dissolving 25 mg PVC, 50 mg DOS, 22 mg TDDA-TPFPhB and 3 mg NaTPFPhB in 1 mL freshly distilled of tetrahydrofuran (THF) (Sigma-Aldrich, St. Louis, MO, USA). This membrane cocktail was used to spin-coat the surface of a 3 mm diameter glassy carbon (GC) electrode (Model MF-2012, BASi, West Lafayette, IN, USA). Before the membrane deposition, the surface of the GC electrode was polished on wet microcloth pads using Al_2_O_3_-based slurry (Mager Scientific, Dexter, MI, USA) with grain sizes of 1, 0.3 and 0.05 μm [31]. For spin-coating, the electrode was secured in the three-jaw chuck of a Dewalt 20V MAX drill press (Baltimore, MD, USA) and dipped into a PVC membrane cocktail. After the removal of the electrode from the cocktail, it was rotated for 60 s at 2000 rpm and left in an upright position until the complete evaporation of THF. This protocol resulted in 1–3 µm thick PVC membrane-coating on the electrode surface. For spin-coating the CHI Model 131 electrode, which was used in the CHI Model 130 flow cell, a KW-4 spin coater (SETCAS LLC, San Diego, CA, USA) was employed at 1650 RPM rotation rate. While the GC electrode was rotated, 50 µL of the membrane solution was dropped on the center of the electrode. The GC electrode was rotated for 3 min and then was left for 3 h for complete THF evaporation.

### 2.2. Batch Measurements with Linear Sweep Voltammetry and Chronoamperometry 

Linear sweep voltammetry (LSV), and chronoamperometry (CA) experiments using the PVC membrane-coated GC electrodes were performed in pH ~7.2 phosphate buffered saline (PBS) buffer solution with 0.1 mol/L potassium chloride (KCl) (Sigma-Aldrich, St. Louis, MO, USA) or whole blood, with ~0.17% Ethylenediaminetetraacetic acid (EDTA) as anticoagulant, using a GC rod and a single junction silver/silver chloride (Ag/AgCl) 1 mol/L KCl reference electrode as the counter and reference electrodes, respectively. The PBS buffer (pH ~7.2) was prepared as a mixture of 0.1 mol/L KH_2_PO_4_ (Sigma-Aldrich, St. Louis, MO), 0.1 mol/L K_2_HPO_4_ (Sigma-Aldrich, St. Louis, MO), 0.1 M KCl, and 0.045 mol/L NaOH (Sigma-Aldrich, St. Louis, MO). The PBS buffer solutions were spiked with stock solutions of ascorbic acid (AA)(Acros Organics, a Thermo Fisher Scientific Subsidiary, Waltham, MA, USA), p-acetamino phenol (APAP) (Sigma-Aldrich, St. Louis, MO, USA) and the individual drugs. The LSV scans were performed between 0 and 1.4 V at 0.1 V/s scan rate. The CA experiments were performed at the peak potentials determined in the LSV experiments for the individual drugs. The calibration curves were recorded by spiking a PBS background electrolyte or whole blood by stock solutions. For the voltammetric measurements, a CH Instruments (Austin, TX, USA) model CHI 842B potentiostat was used with CH Instruments software version 18.01.

### 2.3. Chronoamperometry in a Continuous Flow Analysis Mode

For the continuous flow analysis (CFA) measurements, a CH Instruments model CH130 thin layer flow cell was used. In the thin layer cell, the PVC membrane-coated GC working electrode was used in combination with an Ag|AgCl|3.0 mol/L sodium chloride (NaCl) reference electrode and a stainless steel counter electrode. The cell was connected with a Gilson Minipuls 3 peristaltic pump and Hamilton modular valve positioner. The standard solutions in 0.1 mol/L, pH ~7.2 PBS were transported by 0.5 mL/min flow rate. 

## 3. Results

### Voltammetric Measurement of Highly Lipophilic Molecules

In voltammetric analysis, the modification of the working electrode surface may be motivated by various reasons, e.g., to improve the selectivity, sensitivity and detection limit of the method [33,34]. By coating a working electrode surface with an organic film, e.g., a plasticized PVC membrane, analyte molecules spontaneously partition into the membrane. For highly lipophilic analytes, it means that the analyte concentration in the membrane can be orders of magnitudes larger than in the aqueous sample. Since the analytically relevant signal of the membrane-coated sensor is proportional to the analyte concentration in the membrane, the attainable detection limit with the membrane-coated sensor is expected to be much lower compared to measurements with an unmodified working electrode. The extension of the detection limit towards lower concentrations can be estimated from the current ratio (or slope/sensitivity ratio), which is expected at a given concentration with or without coating the working electrode surface. The correlation between the measured current and the concentration in linear sweep voltammetry (LSV) using a macroelectrode is described by Equation (1) (Randles–Sevcik equation) and for micro disc electrodes at steady state by Equation (2). The corresponding current ratios are provided by Equations (3) and (4), respectively.

(1) $$i_{p,w}=\left(2.69\times 10^{5}\right)n^{3/2}{{AD}_{w}}^{1/2}c_{w}v^{1/2}$$

(2) $$i_{L,w}=4nFD_{w}rc_{w}$$

(3) $$\frac{i_{p,m}}{i_{p,w}}=\frac{{D_{m}}^{1/2}c_{m}}{{D_{w}}^{1/2}c_{w}}$$

(4) $$\frac{i_{L,m}}{i_{L,w}}=\frac{D_{m}c_{m}}{D_{w}c_{w}}$$

where $i_{p,w}$,$\;i_{p,m}$ and $i_{L,w}$, $i_{L,m}$ are the peak or limiting currents in amperes, respectively, n number of electrons involved in the electrochemical reaction, F (C/mol) is the Faraday number, A $\left({\mathrm{cm}}^{2}\right)$ is the surface area of the electrode, r (cm) is the radius of the micro disc electrode, $D\;\left({\mathrm{cm}}^{2}/s\right)$ is the diffusion coefficient, c $\left(\mathrm{mol}/{\mathrm{cm}}^{3}\right)$ is the concentration and v (V/s) is the scan rate. The subscripts w or m indicate the currents, concentrations, and diffusion coefficients in water or in the membrane phase. 

The analyte concentration in the membrane can be estimated using the octanol/water partition coefficient ($P_{o/w}$):

(5) $$P_{o/w}=\frac{c_{o}}{c_{w}}$$

where $c_{o}$ and $c_{w}$ are the concentrations of an analyte in n-octanol and water. As a consequence of the difference in the current versus concentration relationship for macro and microelectrodes (Equations (1) and (2)), the combination of Equation (5) with Equations (3) and (4) shows a significant difference in the expected signal amplification with a membrane-coated macro (Equation (6)) or micro (Equation (7)) electrode. Related to Equations (6) and (7), it must be emphasized that for more realistic estimates of the signal amplification, $P_{o/w}$ should be replaced by $P_{m/w}$, the membrane-water partition coefficient.

(6) $$\frac{i_{p,m}}{i_{p,w}}=\frac{D_{m}{}^{1/2}c_{m}}{D_{w}{}^{1/2}c_{w}}=P_{o/w}\sqrt{\frac{D_{m}}{D_{w}}}$$

(7) $$\frac{i_{L,m}}{i_{L,w}}=\frac{D_{m}c_{m}}{D_{w}c_{w}}=P_{o/w}\frac{D_{m}}{D_{w}}$$

As an example, Equations (6) and (7) were applied to calculate the expected signal amplification for a lipophilic and a hydrophilic drug. The highly lipophilic anesthetic drug propofol ($logP_{o/w}\left(Propofol\right)\approx 3.8$) and p-acetamino phenol (APAP) ($logP_{APAP}\approx 0.31$), a potential interfering compound during propofol measurement, were selected as characteristic examples. As a diffusion coefficient in the aqueous solution, $D_{w}\approx 7\times 10^{-6}{\mathrm{cm}}^{2}/s$ was used [35]. For the diffusion coefficients in the membrane, $D_{m}=2\times 10^{-8}$ ${\mathrm{cm}}^{2}/s$ and $D_{m}=2\times 10^{-7}$ ${\mathrm{cm}}^{2}/s$ were used [36,37]. These values were reported as ionophore diffusion coefficients in the ion-selective membranes [36] or calculated from the scan rate dependence of the peak current (Equation (1)) [37] in plasticized PVC membranes with 1 to 2 PVC to plasticizer ratio. The results of these calculations are summarized in Table 1. 

As shown in the Table 1, a remarkable signal amplification can be achieved ($i_{p,m}/i_{p,w}\gg 1$) with highly lipophilic drugs (analyte with large $P_{o/w}$), while the signal for hydrophilic drugs is reduced compared to an uncoated electrode. The amplification of the analyte signal increases the signal to noise and signal to background current ratios. Thus, it can be assumed that the signal amplification is inversely proportional to the detection limit. Consequently, detection limits in the lower nanomolar concentrations may be attainable with voltammetric methods utilizing organic membrane-coated working electrodes. However, there are significant differences in the expected signal amplification with macro and microelectrodes. While microelectrodes have unique advantages for voltammetric measurements in highly resistive media, like the plasticized polymeric membranes (small IR potential drop), the detection limit using LSV is expected to be better with the membrane-coated macroelectrode. The difference is related to the influence of the diffusion coefficient on the current signal, i.e., $\sqrt{D_{m}}$ for macroelectrodes but $D_{m}$ for microelectrodes.

On the other hand, the discrimination against hydrophilic interferences is expected to be the same with the membrane-coated macro and microelectrode. It depends only on the partition coefficients of the drugs. Using the octanol/water partition coefficient of propofol and APAP, we get:

$$discrimination=\frac{P_{o/w}^{propofol}}{P_{o/w}^{APAP}}=\frac{6309}{2.04}=3090$$

The discrimination can also be determined experimentally from the $i_{p,m}/i_{p,w}$ or calibration slope ratios for the analyte and the interfering compound. Using the data in columns 2 and 4 of Table 1 provides the same results for both the macro and the microelectrode:

$$discrimination=\frac{{\left(\frac{i_{p,m}}{i_{p,w}}\right)}_{propofol}}{{\left(\frac{i_{p,m}}{i_{p,w}}\right)}_{APAP}}={\left(\frac{337.26}{0.109}\right)}_{macroelectr.}={\left(\frac{18.03}{0.0058}\right)}_{microelectr.}=3090$$

**Figure 2:**
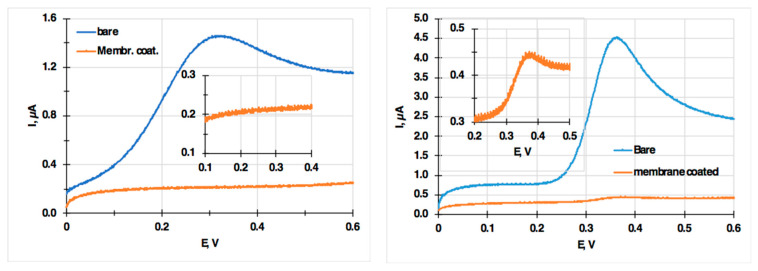
Linear sweep voltammograms recorded with a bare and a membrane-coated electrode in 49.8 µmol/L ascorbic acid (AA) (**left**) and 117.6 µmol/L p-acetamino phenol (APAP) (**right**) solutions. The insets show the membrane-coated sensor signal with much higher resolution in the current scale. The PVC membrane composition is 25% PVC, 50% bis(2-ethylhexyl)sebacate (DOS), 22% tetradodecylammonium tetrakis(pentafuorophenyl)borate (TDDA-TPFPhB) and 3% sodium tetrakis[3,5bis(trifluoromethyl) phenyl] borate (NaTFPhB).

In Figure 2, linear sweep voltammograms recorded with a bare and a membrane-coated electrode in ascorbic acid (AA) and p-acetamino phenol (APAP) solutions are shown to demonstrate the effectiveness of the organic membrane coating for minimizing the interference of electrochemically active, hydrophilic compounds on the voltammetric signal. As seen in the insets, 49.8 µmol/L AA did not result in any measurable current signal with the PVC membrane-coated electrode, while in the 117.6 µmol/L solution of APAP solutions, the current signal was about 20 times lower than with the bare electrode. The difference between AA and APAP is related to the difference in their partition coefficients [38]: logP_AA_ = −1.84 and logP_APAP_ = 0.31.

**Figure 3:**
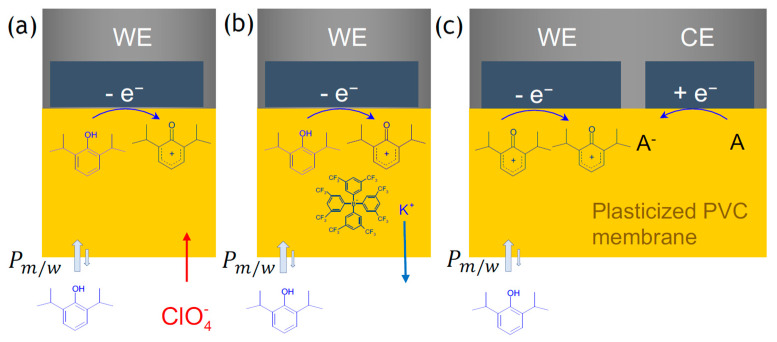
Three different variations of the processes during voltammetric measurements of propofol with the PVC membrane modified electrode. In all three examples, propofol preferentially partitions into the membrane, and during its electrochemical oxidation, propofol positively charged intermediates are generated. The electroneutrality in the membrane is sustained through (**a**) the uptake of hydrophobic counterions, e.g., ($ClO_{4}^{-}$) from the solution; (**b**) the release of positively charged counterions of the cation exchanger (e.g., K^+^) incorporated in the membrane; (**c**) the generation of negatively charged species on the counter electrode. In this scenario, both the working (WE) and counter (CE) electrodes are coated with the membrane.

In Figure 3, the processes during voltammetric measurements of propofol with the PVC membrane-modified electrode are summarized. The plasticized PVC membrane used for coating the GC electrode surface was similar to membranes used in ionophore-based ion-selective electrodes and for ITIES measurement of charged molecules [19,21,23]. However, when these membranes are used in voltammetric measurements, they must also contain a lipophilic background electrolyte, e.g., tetradodecylammonium tetrakis(pentafluorophenyl) borate (TDDATPFPhB). These membranes are labeled as “liquid membranes” due to their very high plasticizer content (up to 86%) [39] in which the diffusion coefficients are similar to viscous solutions. Highly lipophilic compounds like propofol preferentially partition in the membrane plasticizer in which the voltammetric measurement is performed. The enrichment of the analyte in the plasticized PVC membrane is controlled by the partition coefficient of the analyte. The logarithm of the octanol/water partition coefficient ($logP_{o/w}$) of propofol is between 3.83 and 4.15, i.e., the concentration of propofol is about 10,000 times higher in the membrane where the electrochemical reaction occurs than in the aqueous sample solution. At the same time, the hydrophilic interferences, such as ascorbic acid (logP_AA_ = −1.84) [38] and p-acetamino phenol (logP_APAP_ = 0.31) [38], are minimally extracted into the membrane phase, i.e., their concentration generally is negligible in the membrane, which explains the LSVs in Figure 2.

**Figure 4:**
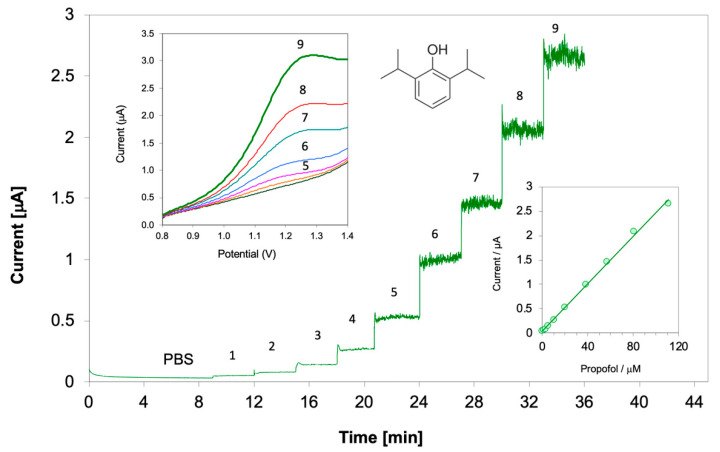
Chronoamperometric response of a PVC-membrane-coated glassy carbon (GC) electrode upon spiking the phosphate buffered saline (PBS) background electrolyte with propofol standard solutions. The concentrations in the PBS solution labeled 1 to 9 are 1.25, 2.5, 4.98, 9.9, 19.6, 38.4, 56.6, 80.5 and 111.1 μmol/L. The inset in the lower right is the calibration curve constructed from the chronoamperometric currents. The inset in the upper left corner shows linear sweep voltammograms recorded in PBS background solution and in PBS with propofol concentrations between 9.9 and 111.1 μmol/L. Membrane composition 25.5% PVC, 50.9% 2-nitrophenyl octyl ether (o-NPOE), 21.2% tetradodecylammonium tetrakis(pentafluorophenyl) borate (TDDATPFPhB), 2.4% sodium tetrakis[3,5bis(trifluoromethyl) phenyl] borate (NaTFPhB).

Figure 4 shows the response of the membrane-coated propofol sensor during calibrations by chronoamperometry and linear sweep voltammetry. The attainable detection limit of the membrane-coated propofol sensor was calculated from the standard deviation of the noise ($SD_{bkg}$) in the background electrolyte during linear sweep voltammetric and chronoamperometric measurements and the slopes of the corresponding calibration curves (S) using the formula:

$$LOD=3\times SD_{bkg}/S$$

In addition, we assessed the resolution of the propofol measurement using the residual mean standard deviation (RMSD) of the data points around the line fitted to the data points of the calibration curve, instead of the standard deviation of the background noise. The resolution is defined as a minimum concentration difference between two solutions essential for their quantitative determination.

Resolution=3×RMSD/S

The calculated values in the presence of different background electrolytes and using calibration data points recorded in narrower or broader concentration ranges are summarized in Table 2 [31,40,41].

As shown in Table 2, by coating the GC working electrode surface with the plasticized PVC film, the detection limit in propofol measurements can be extended below the lower limit of the therapeutic concentration range (1 and 60 μmol/dm^3^). The interference induced by electrochemically oxidizable species are negligible (AA) or small (APAP), even if those are at the maximum of their therapeutic concentrations. From the data, it appears that by optimizing the membrane composition, i.e., the selection of the membrane plasticizer, lipophilic background electrolyte and ion-exchanger, and their concentration in the membrane, both the selectivity and the detection limit of the sensor may be improved. Finally, it is important to note that in contrast to aqueous electrolyte solutions, during the chronoamperometric determination of propofol with the PVC membrane-coated sensor, no electrode fouling is observed [31].

Based on Equations (6) and (7), the signal amplification of the organic film (PVC membrane-coated) voltammetric sensor is directly proportional with the partition coefficient of the target analyte between the membrane and aqueous phase, i.e., the lowest detection limits are expected during the voltammetric determination of the most lipophilic compounds. On the other hand, the selectivity of the membrane modified electrode is controlled by the partition coefficient ratio of the analyte and the interfering compound. Consequently, the voltammetric determinations with the membrane modified electrodes are most desirable for tasks requiring the measurement of sub-micromolar concentrations of lipophilic analytes in the presence of large excess hydrophilic interferents. A list of electrochemically active target analytes (antidepressant and immunosuppressant drugs) are summarized in Table 3. All these drugs are highly lipophilic with therapeutic blood concentration ranges below 1.0 μmol/L, which could be measured in the presence of up to 1.3 mmol/L APAP and 0.06 mmol/L AA. 

**Figure 5:**
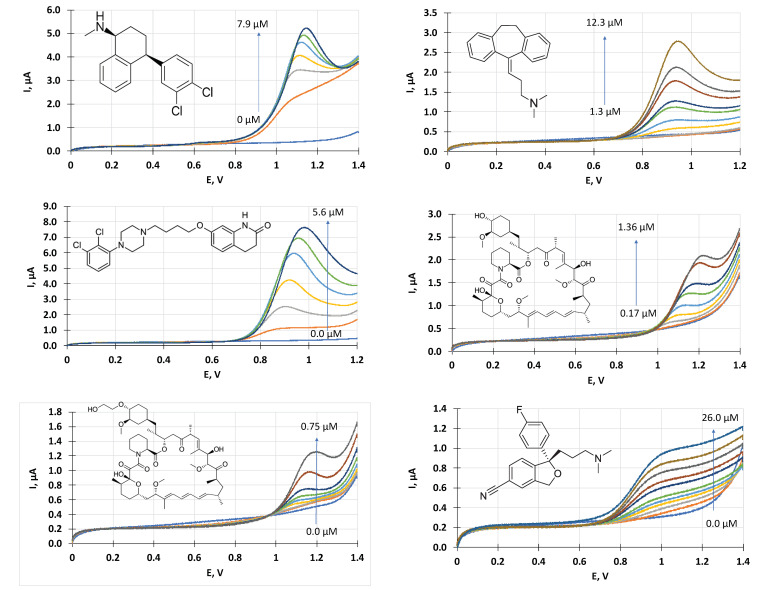
Linear sweep voltammograms recorded in sertraline (**upper left**), amitriptyline (**upper right**) and aripiprazone (**middle left**), sirolimus (**middle right**), everolimus (**lower left**) and citalopram (**lower right**) solutions. The concentration ranges are indicated in the individual pictures.

**Figure 6:**
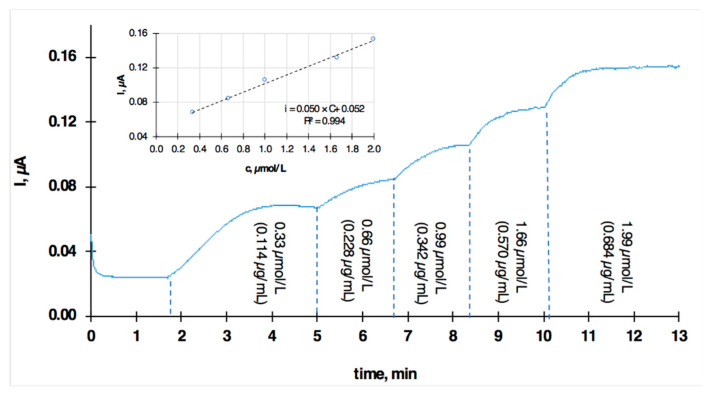
Chronoamperometric transients recorded with the plasticized PVC membrane-coated GC electrode in PBS solution of different sertraline concentrations. Applied potential: 1.0 V.

**Figure 7:**
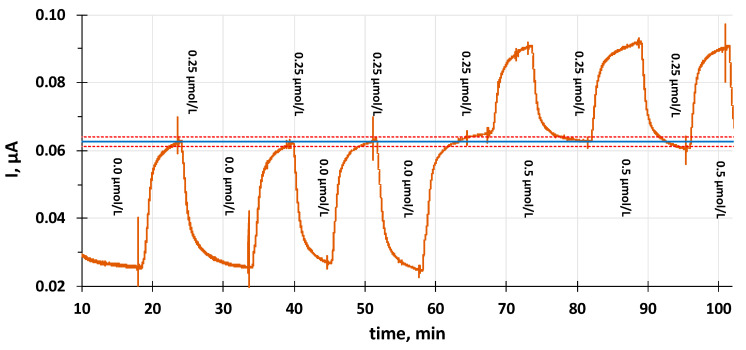
The reproducibility of the chronoamperometric measurements in a continuous flow mode of measurement of amitriptyline with the PVC membrane-coated GC electrode in PBS solution. Applied potential: 0.95 V. The standard solutions are switched between 0 and 0.25 µmol/L and 0.25 and 0.5 µmol/L in the PBS background. The blue line represents the mean value of the current in 0.25 µmol/L amitriptyline and the red dotted lines represent ±1 standard deviation range of the data around the mean.

LSVs recorded with membrane-coated glassy carbon (GC) electrodes in sertraline, amitriptyline, aripiprazole, sirolimus, everolimus and citalopram solutions of different concentrations are shown in Figure 5. The peak currents of the LSVs were used to construct calibration curves for the individual drugs. Calibration curves were also recorded using a chronoamperometric measurement protocol by applying the drug-specific potential values to the PVC membrane-coated electrode. The chronoamperometric transients recorded during the calibration of the PVC membrane-coated electrode in sertraline solutions and the corresponding calibration curve is shown in Figure 6 as an example. Based on the calibration curves recorded with linear sweep voltammetry and chronoamperometry, we calculated the LOD and resolution of the determinations for all the drugs. The results of the calculations are summarized in Table 4. As shown in Table 4, both types of measurement methods result in sub-micromolar detection limits, and in the case of amitriptyline, even in whole blood. In agreement with the expectations, both the LOD and resolution values are better in chronoamperometric experiments. The detection limit and the resolution of the chronoamperometric method could be further improved by performing the measurements in a flow through manifold (not shown). The reproducibility of the chronoamperometric measurements in a continuous flow mode of measurement is shown in Figure 7. 

**Figure 8:**
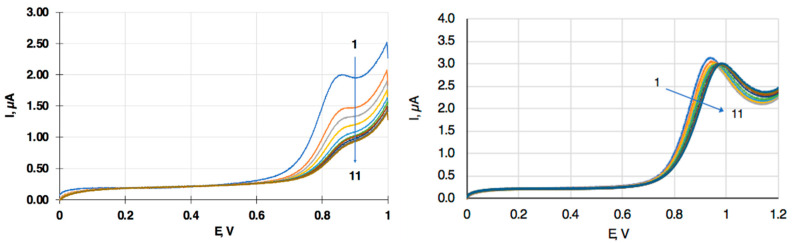
Repeated LSVs of a bare (**left**) and PVC membrane-coated (**right**) GC electrode in 50 µmol/L (bare, **left**) and 10 µmol/L (membrane-coated, **right**) amitriptyline containing solutions.

Using a plasticized PVC membrane-coated glassy carbon (GC) electrode for the measurement of lipophilic drugs also prevented electrode fouling, which is commonly experienced with the uncoated electrode (Figure 8).

## 4. Conclusions

The simple modification of voltammetric working electrodes by coating their surfaces with a thin film of highly plasticized PVC membrane (an organic film) opens up the possibility of monitoring highly lipophilic drugs continuously in biological samples. In general, PVC membrane coating prevents electrode fouling and permits the quantification of analytes in sub-micromolar concentrations even in the presence of a large excess of electrochemically active, hydrophilic interfering compounds. The improvement in the sensitivity of the voltammetric measurements with the PVC membrane-coated working electrode, compared to measurements with conventional working electrodes without coating, is a function of the increased concentration and decreased diffusion coefficient ($\sqrt{D}$ or D) of the analyte in the membrane in which the voltammetric measurement is performed. For the lipophilic analytes discussed in this paper, the concentration increase is estimated between 10^4^ and 10^7^ (from $logP_{o/w}=4$ to $logP_{o/w}=7$). At the same time, the diffusion coefficients in the highly plasticized PVC membranes are about 2–2.5 orders of magnitude smaller than in aqueous solutions. As a consequence, a three orders of magnitude signal amplification and a three orders of magnitude improvement in the detection limit can be realized. The selectivity improvement with the membrane-coated voltammetric sensor is a function of the partition coefficients for the analyte and the interfering compound. Consequently, hydrophilic analytes at their therapeutic concentration, e.g., ascorbic acid and p-acetamino phenol, have no or only negligible effects on the plasticized PVC membrane-coated sensor signal. 

## Figures and Tables

**Table 1:** Peak current ratios ($i_{p,m}/i_{p,w}$) which would be expected if linear sweep voltammetry (LSV) experiments were performed with or without coating the working electrode surface with a plasticized PVC membrane in solutions of the same concentration.

	${\left(i_{p,m}/i_{p,w}\right)}_{propofol}$		${\left(i_{p,m}/i_{p,w}\right)}_{APAP}$	
	$D_{m}=2\times 10^{-8}\;{\mathrm{cm}}^{2}/s$	$D_{m}=2\times 10^{-7}\;{\mathrm{cm}}^{2}/s$	$D_{m}=2\times 10^{-8}\;{\mathrm{cm}}^{2}/s$	$D_{m}=2\times 10^{-7}\;{\mathrm{cm}}^{2}/s$
Macroelectrode, Equation (6)	337	1066	0.11	0.35
Microelectrode, Equation (7)	18.0	180	0.006	0.06

**Table 2:** Detection limit (LOD) and resolution values calculated for the propofol sensor from triplicate chronoamperometry (CA) studies in different background electrolytes. The background electrolytes are prepared in PBS containing 3 mmol/L ascorbic acid (AA), 1 mmol/L p-acetamino phenol (APAP), 5% w/v bovine serum albumin (BSA) or a combination of all three of these potential interferents (mix).

Membrane Composition *	Background	Concentration Range † [μmol/L]	LOD [μmol/L]	Resolution [μmol/L]
o-NPOE plasticizer	PBS	0–56.6	0.03 ± 0.01	1.1 ± 0.2
	3 mmol/L AA	0–56.6	0.05 ± 0.05	2.0 ± 1.0
	1 mmol/L APAP	0–56.6	0.08 ± 0.02	4.6 ± 0.9
	5% BSA	5–56.6	2.2 ± 3.1	14 ± 2
	mix	2.5–109.8	0. 5 ± 0.4	28 ± 5
DOS plasticizer	PBS	0–56.6	0.12 ± 0.05	4.3 ± 0.4
	mix	0–56.6	3.0 ± 0.3	4.5 ± 2.3

* The composition of the PVC membranes are 25.5% PVC, 50.9% 2-nitrophenyl octyl ether (o-NPOE), 21.2% tetradodecylammonium tetrakis(pentafluorophenyl) borate (TDDATPFPhB), 2.4% sodium tetrakis[3,5bis-(trifluoromethyl) phenyl] borate dihydrate (NaTFPhB) and 25.1% PVC, 49.9 bis(2-ethylhexyl)sebacate (DOS), 22.6% TDDATPFPhB and 2.4% potassium tetrakis-(pentafluorophenyl) borate (KTPFPhB). † The slope and RMSD value of the calibration curve was calculated from data points recorded in the concentration range.

**Table 3:** List of highly lipophilic, electrochemically active analytes (antidepressant drugs) with sub-micromolar therapeutic concentration ranges as potential targets for the development of voltammetric methods based on organic film-coated working electrodes. The log *p* values are generally from DRUGBANK (www.drugbank.ca). Experimentally determined values are provided with the source. SSRI stands for selective serotonin reuptake inhibitors.

Sertraline (Zoloft) Antidepressant SSRI	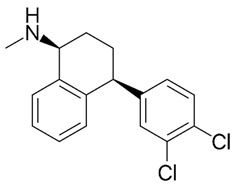	log P = 5.06–5.15 MW: 306.229 g/mol Concentration range: 30–200 ng/mL 0.1–0.65 µmol/L
Amitriptyline (Elavil) Antidepressant	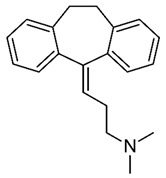	log P = 4.81–5.1 log P_exp_ = 4.92 [42] MW: 277.403 g/mol Concentration range: 75–175 µg/L 0.27–0.63 µmol/L
Aripiprazole (Abilify) Antipsychotic	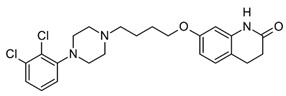	log P = 4.9–5.21 MW: 448.385 g/mol Concentration range: 150 and 300 ng/mL 0.33–0.67 µmol/L
Sirolimus (Rapamycin) Immunosuppressant	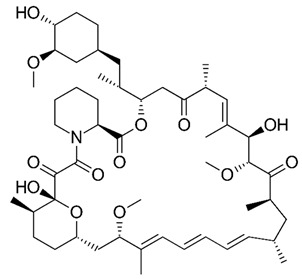	log P = 4.8–7. 5 MW: 914.2 g/mol Concentration range: 4–20 ng/mL 4.4–21.9 nmol/L
Everolimus (Zortress) Immunosuppressant	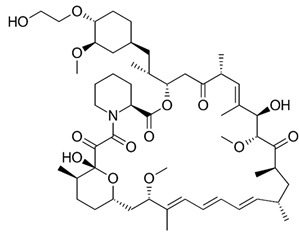	log P = 5.0–7.4 MW: 958.240 g/mol Concentration range: 3–8 ng/mL 3.1–8.3 nmol/L
Citalopram (Celexa) Antidepressant SSRI	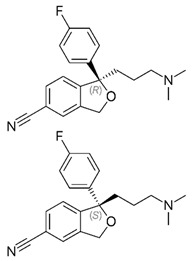	log P = 3.6–3.8 MW: 324.392 g/mol Concentration range: 50–100 ng/mL 0.15–0.3 µmol/L

**Table 4:** Detection limit (LOD), resolution of the plasticized PVC membrane-coated GC electrode for amitriptyline, sertraline, aripiprazole, sirolimus, everolimus and citalopram.

Analyte	Method	Matrix	LOD µmol/L	Resolution µmol/L
Amitriptyline	LSV	PBS Whole blood	0.09 ± 0.05 (*n* = 3) 0.07 ± 0.03 (*n* = 2)	0.60 ± 0.2 (*n* = 3) 0.09 ± 0.03 (*n* = 2)
	CA	PBS Whole blood	0.01 ± 0.03 (*n* = 5) 0.03	0.05 ± 0.02 (*n* = 3) 0.07
Sertraline	LSV	PBS	0.13	0.75
	CA	PBS	0.003	0.12
Aripiprazone	LSV	PBS	0.02	0.43
	CA	PBS	0.009	0.07
Sirolimus	LSV	PBS	0.02	0.07
	CA	PBS	0.003	0.02
Everolimus	LSV	PBS	0.01	0.05
	CA	PBS	0.02	0.04
Citalopram	LSV	PBS	0.6	0.7
	CA	PBS	0.008	0.08
